# L-type calcium channel modulation reveals the relationship between neuronal synchrony and hyperactivity in the inferior colliculus following noise-induced hearing loss

**DOI:** 10.1038/s41598-025-29536-8

**Published:** 2025-12-09

**Authors:** Selin Yalcinoglu, Rod D. Braun, Ammaar Wattoo, Aaron K. Apawu, Rasheed Alrayashi, Avril Genene Holt

**Affiliations:** 1 Ophthalmology, Visual and Anatomical Sciences, School of Medicine, Wayne State University, 550 East Canfield, Detroit, MI 48201 USA; 2 John D. Dingell, Veterans Affairs Medical Center, Detroit, MI 48201 USA; 3 Department of Chemistry and Biochemistry, University of Northern Colorado, Greeley, CO 80639 USA

**Keywords:** Noise-induced hearing loss, ABR (auditory brainstem response), Gap inhibition of acoustic startle reflex, Neuronal activity, Inferior colliculus, Cochlea, Neuroprotection, Calcium channel blockers, Auditory system, Auditory system, Cochlea, Midbrain

## Abstract

Previous studies have established the protective effects of calcium channel inhibition on the peripheral auditory system in response to noise exposure. While these studies implicate L-type calcium channels (LTCCs) in noise-generated dysfunction in the auditory periphery, contributions of LTCCs to noise-induced central dysfunction remains unclear. To begin to elucidate the roles of LTCCs in hearing, peripheral and central auditory function were assessed longitudinally after LTCC inhibition. Neuronal synchrony and activity were assessed by analyzing wave I (peripheral) and wave V (central) auditory brainstem responses (ABRs). Just prior to a noise exposure resulting in a temporary shift in hearing thresholds, rats were administered verapamil (LTCC blocker) or saline. Verapamil administration prevented the noise-induced decrease in ABR wave I and V amplitudes. Interestingly, when non-noise exposed animals were administered verapamil, wave V amplitude decreased, suggesting that LTCCs are critical for neuronal synchrony in the inferior colliculus. The inferior colliculus mediates gap inhibition of the acoustic startle reflex (giASR). Following noise exposure, giASR was enhanced, but the enhancement was not prevented by LTCC antagonism. These results suggest that while LTCCs are necessary for auditory-related synchronous activity, these channels do not directly contribute to noise-induced hyperactivity in the inferior colliculus.

## Introduction

Hearing loss affects approximately 48 million Americans, with more than half having lasting damage due to excessive noise exposure^1^. Hearing loss can impact quality of life, cognition, and mental health with increased suicidal ideations^2^. Individuals whose occupational and recreational activities involve noisy environments are at higher risk for permanent or temporary hearing impairment (e.g., factory workers, military personnel, dental hygienists, musicians, or athletes)^1,3^.

Regardless of the severity of the noise-induced hearing loss (NIHL), there can be sustained changes in peripheral and central auditory-related function. Additionally, exposure to loud noise has been associated with synaptopathy (a loss of synaptic connection between inner hair cells and the auditory nerve in the periphery), and increased neuronal activity in central auditory brain regions, such as the inferior colliculus^4–6^.

Neuronal activity throughout the auditory pathway is primarily regulated by free calcium entering neurons via voltage-gated calcium channels, which trigger neurotransmitter release^7–9^. The L-type calcium channel family consists of four distinct subtypes (Cav1.1-Cav1.4), with Cav1.2 and Cav1.3 channels playing critical but distinct roles in auditory processing^10–13^. Cav1.3 channels are essential for synaptic transmission at inner hair cell (IHC) ribbon synapses in the cochlea^14^. These channels cluster at the presynaptic active zones of IHCs, where they mediate calcium influx that triggers glutamate release onto auditory nerve fibers^11–13,15,16^. In contrast, Cav1.2 channels are widely distributed in postsynaptic neurons throughout the brain, including the inferior colliculus, where they regulate synaptic plasticity and gene transcription^10,17–19^.

The afferent auditory pathway exhibits a distinct calcium channel subtype organization. At the peripheral level, synaptic transmission relies predominantly on Cav1.3 channels at IHC-auditory nerve synapses, which show relatively low sensitivity to verapamil and other phenylalkylamine calcium channel blockers^11–13,15,20^. Further upstream in the central auditory pathway, synaptic transmission depends primarily on P/Q-type (Cav2.1) channels, which are insensitive to verapamil^21–23^. These Cav2.1 channels are the predominant calcium channel subtype at auditory brainstem synapses, where they support the high-frequency, reliable synaptic transmission required for temporal coding of acoustic information^21–24^.

Verapamil, a phenylalkylamine L-type calcium channel antagonist, exhibits differential pharmacological selectivity between calcium channel subtypes^20,25–27^. The drug demonstrates markedly higher affinity for Cav1.2 channels compared to Cav1.3 channels, with studies showing 5–tenfold differences in inhibitory potency^20,25,26^. This selectivity profile arises from structural differences in the drug binding pockets between the two channel subtypes. The verapamil-sensitive Cav1.2 channels are present in almost all neurons (predominantly postsynaptic) and are crucially involved in activity-dependent synaptic plasticity and calcium-dependent gene transcription^10,19,27^.

The effects of increased calcium accumulation due to excessive neuronal activity can range from ototoxicity and cell death to increased spontaneous neuronal activity and have been linked to multiple auditory-related disorders^28–32^. Thus, regulation of calcium channel function might represent a potential therapeutic approach to protect from both peripheral and central contributions to NIHL. Previous studies in rodents have used various calcium channel antagonists to reduce the impact of noise-induced permanent threshold shifts in the periphery with some success^18,33–36^. However, there remains a significant gap in knowledge regarding the effects of calcium channel inhibition on noise-induced synaptic function centrally.

Verapamil, a non-dihydropyridine L-type calcium channel antagonist, has few side effects and readily crosses the blood–brain barrier to target calcium channels centrally.Voltage-gated calcium channel function and calcium mobilization are crucial for normal auditory transmission^14,37–39^. Since noise exposure disrupts spontaneous neuronal activity^17,20,30^, understanding the balance between hyperactivity and synchrony provides insight into noise-related neural manifestations.

Neuronal synchrony refers to the precise temporal coordination of compound action potentials (firing) across a population of neurons in response to auditory stimuli^37^. In contrast, hyperactivity refers to abnormally increased spontaneous or evoked neuronal firing, which can emerge after noise-induced trauma and may occur independently of synchrony^5,40–42^. Neuronal synchrony is crucial for the accurate encoding and transmission of auditory information, particularly for preserving temporal features of complex sounds^16,21,24^. Synchronization is influenced by various factors, including the properties and distribution of different voltage-gated calcium channels, which contribute to both the initiation and regulation of synchronous activity in the auditory pathway.

Therefore, we hypothesize that inhibition of L-type calcium channels will provide protection from noise-induced trauma for both peripheral and central auditory transmission through modulation of the dynamic interplay between synchrony (generated as the result of auditory nerve signaling) and neuronal activity (hypo- vs hyper-activity). The differential sensitivity of Cav1.2 versus Cav1.3 channels to verapamil, combined with their distinct anatomical distributions, predicts that calcium channel inhibition will have contrasting effects on peripheral versus central auditory function.

## Materials and methods

### Subjects and design

Twenty-five young adult male Sprague–Dawley rats (Charles River Laboratory, New Jersey) were randomly divided into four treatment groups. The treatment groups were no noise exposure with saline (n = 6), noise exposure with saline (n = 7), no noise exposure with verapamil (n = 7), and noise exposure with verapamil (n = 7). Approximately 20 min prior to the noise exposure, either verapamil (30 mg/kg) or saline (27 ml/kg, 0.9%) solution was administered intraperitoneally. The noise groups were exposed to a 16 kHz, 106 dB SPL tone for one hour, while the no noise control groups were maintained in ambient noise conditions (60 dB) for an equal amount of time.

All rats were individually housed and maintained at 24 ± 1° C with a 12-h light/dark cycle (lights on at 7 AM). Standard housing conditions with free access to normal rat chow and tap water were provided at Wayne State University’s (WSU) AAALAC-accredited animal facility. Animals were treated in accordance with the Animal Welfare Act. In addition, the DHHS “Guide for the Care and Use of Laboratory Animals” was followed. All procedures were approved by the WSU Institutional Animal Care and Use Committee in accordance with NIH (D16-00,198). This study is reported in accordance with ARRIVE guidelines.

### Noise exposure

Just prior to noise exposure, a single ear of each animal was provided protection by filling the ear canal with a silicone elastomer for sound attenuation during the exposure (Kwik Seal, World Precision Instruments, Sarasota, FL). Awake and freely moving animals were exposed to a 16 kHz tone, 106 dB SPL with a sound pressure level (SPL) of 106 dB generated using software (Daqarta v 10.3) and delivered by overhead speakers for one hour. The silicone plug was removed immediately following the noise exposure. Animals in the no noise group had one ear plugged for one hour but were not exposed to loud noise.

### Auditory brainstem responses (ABRs)

**Fig. 1:**
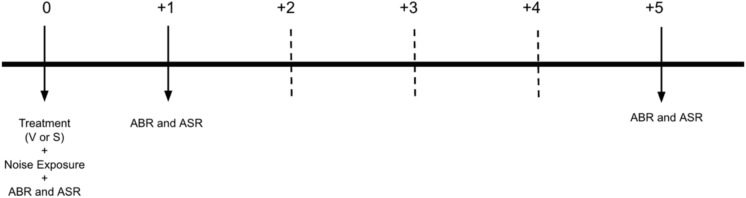
Experimental timeline. Following treatment (verapamil, V or saline, S) and noise exposure, each rat had ABR and ASR tests performed at three different time points: same day after treatment (day 0), one day after treatment (+ 1 day), and five days after treatment (+ 5 days).

ABR analysis evaluated both the amplitudes (waves I and V) and hearing thresholds. Animals were anesthetized with an intramuscular injection of ketamine (75 mg/kg) and xylazine (8 mg/kg). To record the neurological response, subdermal electrodes were placed below the test ear (reference), below the contralateral ear (ground), and at the vertex (active). The assessment was performed at two different frequencies (12 kHz and 20 kHz) at incremental intensities (between 30–85 dB) at three time points (same day, one day, and five days after treatment; Fig. 1). At each intensity amplitudes (μV) and latencies (ms) were calculated. Amplitudes were calculated by subtracting the negative peak from the corresponding positive peak of the response, and latencies were recorded at only the positive peak for each wave (I and V). Amplitudes were assessed first at early (ET, day 0 and 1) and late (LT, day 5) time points and additionally at 0, 1, 5 days after treatment and exposure. Latencies were assessed at only ET and LT points. Hearing thresholds were measured at 12 and 20 kHz, with decreasing increments beginning at 80 dB SPL, until a response was no longer elicited. The lowest intensity that elicited a response was recorded as the threshold.

### Gap inhibition of Acoustic Startle Reflex (giASR)

**Fig. 2:**
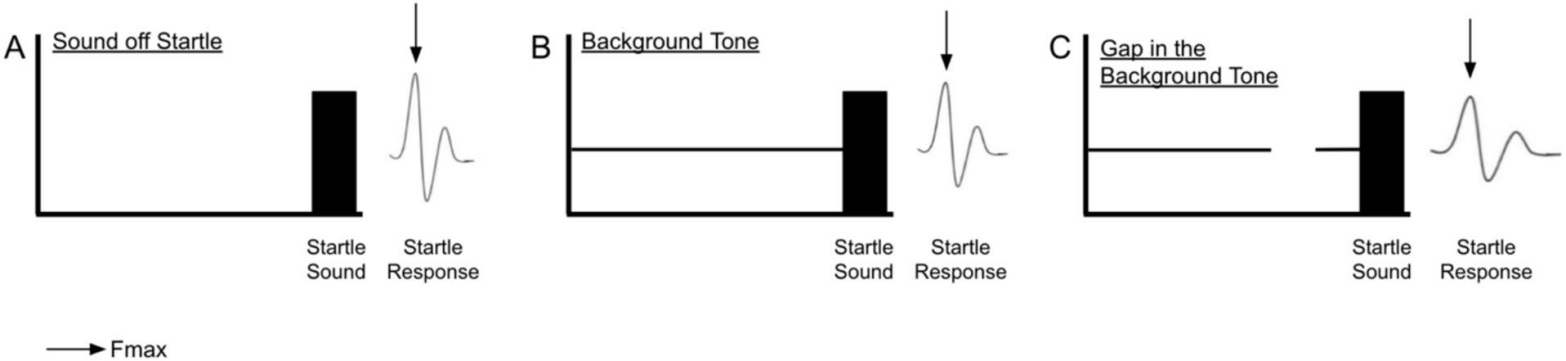
ASR testing paradigm. Gap detection was composed of three types of trials: a silent period followed by a startle sound (solid black bar), i.e., “sound-off startle” or “SOS” trial (**A**), a background tone immediately followed by a startle sound, i.e., “no gap” or “NG” trial (**B**), and a background tone interrupted by a 50 ms silent period before reintroduction of the tone for 50 ms before the startle sound, i.e., “gap” or “G” trial (**C**). In each trial, Fmax (indicated by the arrow), the maximum force exerted during the startle response, was recorded.

giASR testing was performed at six frequencies (4, 8, 12, 16, 20 and 24 kHz) and two intensities (45- and 60-dB SPL). Testing was performed at three time points (same day, one day, and five days after treatment, Fig. 1). The gap inhibition of ASR paradigm was composed of three types of trials (Fig. 2): a silent period followed by a 20 ms startle sound (120 dB broadband noise; Fig. 2A, “sound-off startle” or “SOS” trial), a background tone immediately followed by a startle sound (Fig. 2B, “no gap” or “NG” trial), and a background tone interrupted by 50 ms of silence before reintroduction of the tone for 50 ms before the startle sound (Fig. 2C, “gap” or “G” trial) . In each trial F_max_, the maximum Newton force exerted in response to the startle sound, was recorded. Following the testing, the F_max_ in gap trial to F_max_ in sound-off startle trial (G/SOS) ratio was analyzed.

### Statistical analysis

All data were analyzed using StatView Version 5.0.1.0 (SAS Institute Inc.) and GraphPad Prism version 10.4.1 for MAC (Boston, MA USA). The data points were assessed using Kolmogorov–Smirnov test and was determined to be normally distributed. Group differences were analyzed by analysis of variance (ANOVA) and Tukey/Kramer post-hoc comparison. Significance of a linear regression was determined using a t-test. To determine if the slope of a line was significantly different from 1, an extra sum-of-squares F test was used. P values less than 0.05 were considered significant. All data are expressed as mean ± standard error of the mean.

## Results

### Verapamil does not impact hearing thresholds in normal or noise exposed animals

To assess whether L-type calcium channel inhibition affects baseline auditory sensitivity, hearing thresholds were measured at 12 and 20 kHz across all treatment groups at multiple time points following verapamil administration and noise exposure.

#### Same day of treatment

**Fig. 3:**
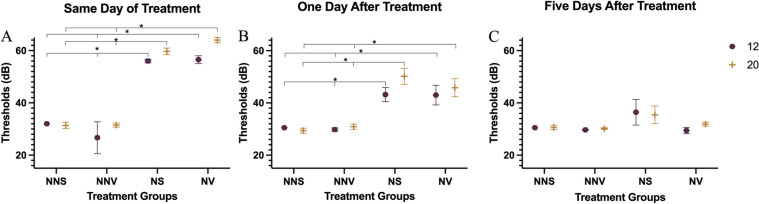
ABR thresholds at 12 and 20 kHz. Three different time points (**A**) same day of treatment, (**B**) one day after treatment, and (**C**) five days after treatment, are shown. Hearing thresholds of each treatment group (no noise saline, no noise verapamil, noise saline and noise verapamil) are shown at 12 kHz (hexagons) and 20 kHz (plus signs). Groups were compared using ANOVA with a post-hoc Tukey–Kramer test, and the error bars indicate the standard error of mean. * Asterisks denote significance at p < 0.05.

On the same day of treatment (Fig. 3A), a significant threshold shift was observed in the noise saline group compared to no noise saline and no noise verapamil groups at 12 kHz (26 dB SPL and 31 dB SPL, respectively, p < 0.05) and at 20 kHz (29 dB SPL, p < 0.05). The noise verapamil group had a significant threshold shift compared to no noise saline and no noise verapamil at 12 kHz (28 dB SPL and 30 dB SPL, respectively, p < 0.05) and at 20 kHz (33 dB SPL, p < 0.05). On average, the ear protected by the silicone elastomer produced a 27 dB attenuation of the threshold shift caused by noise exposure and was not significantly different from the control, no noise saline group.

#### One day after treatment

One day after treatment, thresholds were still elevated in noise groups both at 12 and 20 kHz (Fig. 3B). A significant threshold shift was observed in the noise saline group compared to no noise saline and no noise verapamil groups at 12 kHz (13 dB SPL, p < 0.05) and at 20 kHz (21 dB SPL and 19 dB SPL, respectively, p < 0.05). The noise verapamil group had a significant threshold shift compared to no noise saline and no noise verapamil at 12 kHz (13 dB SPL, p < 0.05) and at 20 kHz (16 dB SPL and 15 dB SPL, respectively, p < 0.05).

#### Five days after treatment

Five days after noise exposure (Fig. 3C) hearing thresholds of both the noise groups returned to baseline with no significant difference compared to no noise groups.

### Verapamil prevents early effects of noise on Wave I input/output (I/O) function

To determine whether L-type calcium channel inhibition protects peripheral auditory nerve fibers from noise-induced dys-synchrony, ABR Wave I amplitudes and latencies were analyzed as indicators of auditory nerve function and inner hair cell synaptic integrity.

#### I/O Function

**Fig. 4:**
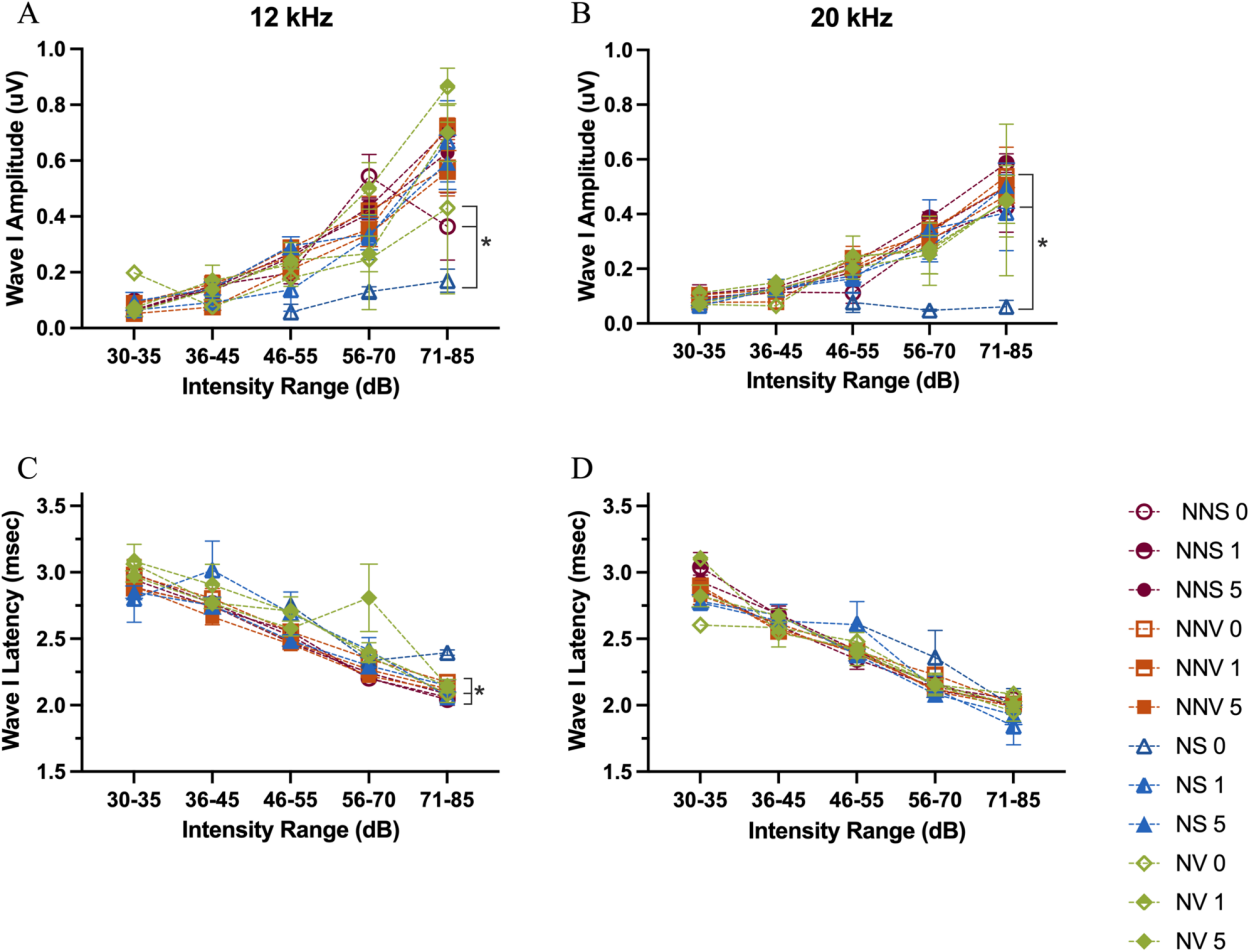
ABR wave I amplitude and latency at 12 and 20 kHz. Wave I input/output function amplitude (**A**-**B**) and latency (**C**-**D**) across 5 intensity ranges are displayed. The wave I amplitudes and latencies were analyzed at three time points across four groups [no noise saline time points (circles: NNS 0—open, NNS 1—half filled, NNS 5—filled), no noise verapamil time points (squares: NNV 0—open, NNV 1—half filled, NNV 5—filled), noise saline time points (triangles: NS 0—open, NS 1—half filled, NS 5—filled), noise verapamil time points (closed diamonds: NV 0—open, NV 1—half filled, NV 5—filled), groups at 12 kHz (A, C) and 20 kHz (B, D). Groups were compared using an ANOVA with a Tukey/Kramer test used for post-hoc comparisons. Error bars indicate standard error of mean. Brackets with asterisks denote significance.

For the 0 time point (day of noise exposure) there was significant decrease in the NS group amplitude compared to NNS and NNV groups at both 12 and 20 kHz (p ≤ 0.0001, Fig. 4A-B). Interestingly, there was also a significant decrease in the NS group amplitude compared to NV at 20 kHz (p = 0.0121, Fig. 4A). Additionally, the NV group is not significantly different compared to either the NNS and NNV groups at 12 or 20 kHz (Fig. 4A-B). By the 1 day time point, there were no significant differences amongst groups. Overall, at the 0 time point following noise exposure, there was a rightward shift of amplitude responses that was prevented by verapamil administration at both 12 and 20 kHz (Fig. 4A-B).

#### Latency

As expected, for each group at each time point Wave I latency at both 12 and 20 kHz (Fig. 4C-D) decreased as the intensity of the tone increased. However, noise disrupted this pattern and for the 5 day time point the NNS and NNV groups showed significantly smaller latencies (p = 0.0113) compared to the NV group at 12 kHz. No significant differences in latency were observed amongst groups at 20 kHz (Fig. 4C-D).

### Verapamil exerts temporal effects on Wave V input/output (I/O) function after noise exposure

To evaluate whether verapamil changes noise-induced central auditory processing and inferior colliculus function, ABR Wave V amplitudes and latencies were examined as measures of I/O function and processing speed in the midbrain at different times following noise exposure.

#### I/O Function

**Fig. 5:**
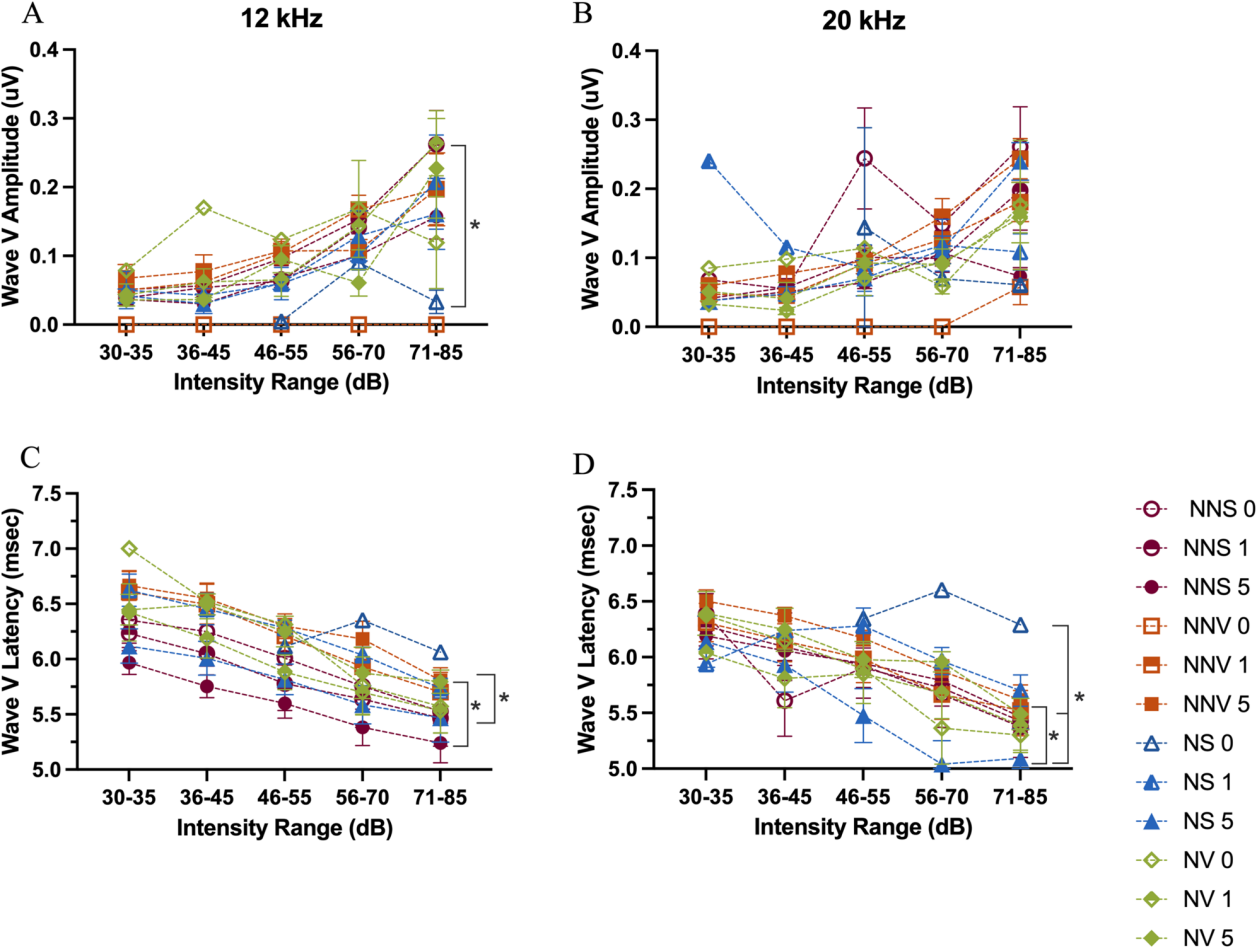
ABR wave V amplitude and latency at 12 and 20 kHz. Wave V input/output function -amplitude (**A**-**B**) and latency (**C**-**D**) across 5 intensity ranges are displayed. The wave V amplitudes and latencies were analyzed at three time points across four groups [no noise saline time points (circles: NNS 0—open, NNS 1—half filled, NNS 5—filled ), no noise verapamil time points (squares: NNV 0—open, NNV 1—half filled, NNV 5—filled ), noise saline time points (triangles: NS 0—open, NS 1—half filled, NS 5—filled), noise verapamil time points (closed diamonds: NV 0—open, NV 1—half filled, NV 5—filled), groups at 12 kHz (A, C) and 20 kHz (B, D). Groups were compared using an ANOVA with a Tukey–Kramer test used for post-hoc comparisons. Error bars indicate standard error of mean. Brackets with asterisks denote significance.

At each time point for the NNS groups (0, 1, and 5 days) Wave V amplitude at both 12 and 20 kHz (Fig. 5B-C) increased as the intensity of the tone increased. Noise disrupted the normal I/O function at 12 kHz with the NNS group showing higher amplitudes when compared to the NS group on the day of noise exposure (p = 0.0001; Fig. 5A). Administration of verapamil reduced, but did not prevent, the effects of noise exposure (Fig. 5A). At 20 kHz the NNS 0 group compared to the NS 0 and NS 1 groups, were not significantly different. The NNV group at the 0 time point showed amplitudes of 0 uV regardless of the intensity tested at 12 kHz and only at the highest intensity tested for 20 kHz (p ≥ 0.080; Fig. 5A-B).

#### Latency

Wave V latency at 12 kHz and 20 kHz, for the NNS groups (Fig. 5C-D) decreased as intensity of the tone increased. Noise disrupted this pattern at the two highest intensity ranges tested. At 12 kHz the NNS group at the 5 day time point remains faster than the NV group (p = 0.0001; Fig. 5C). Also, at 12 kHz the latency for the NNV group at the 5 day time point is significantly delayed compared to the NS group (p = 0.0001; Fig. 5C). For 20 kHz, NS at the 0 time point is significantly delayed compared to NS at the 5 day time point (p = 0.0038; Fig. 5D). The NS group at the 5 day time point is significantly faster compared to the NV group at this same time point (p = 0.0012; Fig. 5D). The NS is also significantly faster than the NNV group at the 5 day time point (p = 0.0001; Fig. 5D).

### Verapamil prevents noise-induced synaptopathy at suprathreshold sound levels

To assess the effect of verapamil on noise-induced cochlear synaptopathy, Wave I amplitudes were compared following suprathreshold stimuli presented across time points in noise-exposed and non-exposed animals treated with verapamil.

#### 12 kHz

**Fig. 6:**
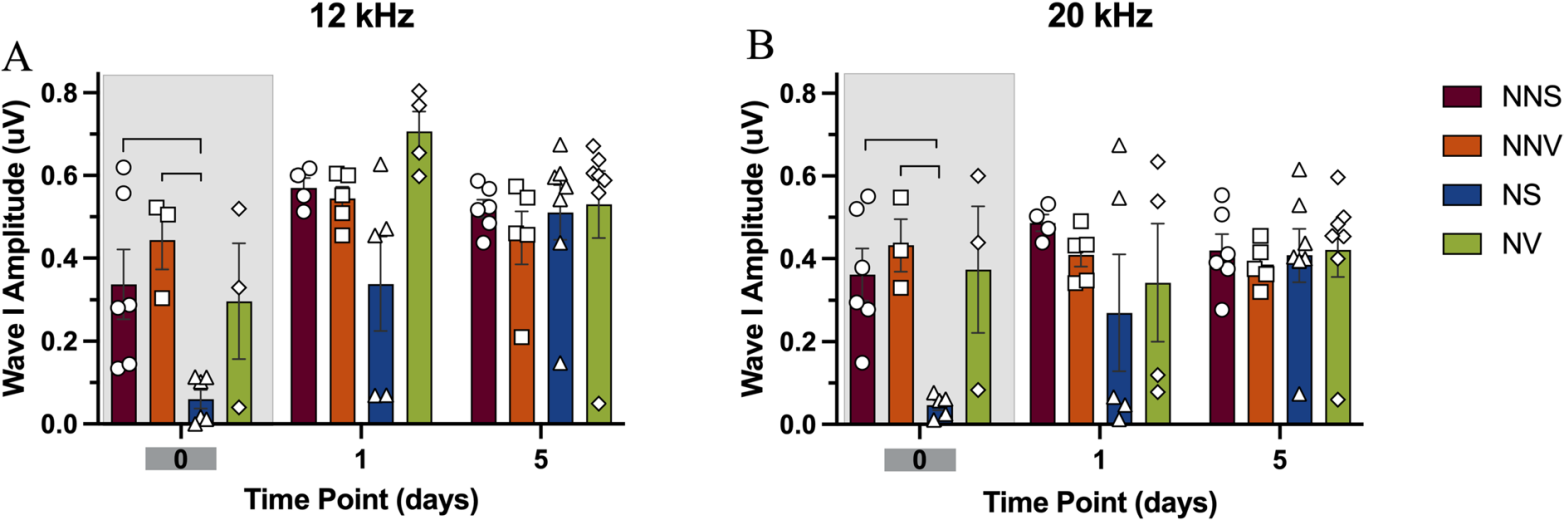
ABR wave I amplitude for auditory brainstem responses at 12 and 20 kHz. Wave I amplitudes on day zero, and one and five days after treatment and noise exposure are displayed. The wave I amplitudes were analyzed for no noise saline (circles—NNS), no noise verapamil (squares -NNV), noise saline (triangles—NS) and noise verapamil (diamonds—NV) groups at 12 kHz (**A**) and 20 kHz (**B**). Groups were compared using ANOVA with a post-hoc Tukey–Kramer test, and the error bars indicate the standard error of mean. Brackets denote significance at p < 0.05. Shaded set of bars indicate the time point at which significant changes were observed.

On day zero of noise exposure there was a significant wave I amplitude reduction in the noise saline group compared to the no noise saline and the no noise verapamil groups (84% and 86% respectively, p < 0.05) at 12 kHz (Fig. 6A). The noise verapamil group was not significantly different when compared to the no noise saline and the no noise verapamil groups. One day after the noise exposure wave I amplitudes returned to normal for the noise saline group with no significant difference between the no noise groups (Fig. 6A). In addition, verapamil did not have any impact on wave I amplitude at later time points (Fig. 6A).

#### 20 kHz

On day zero of noise exposure there was a significant wave I amplitude reduction in the noise saline group compared to the no noise saline and the no noise verapamil groups (77% and 79% respectively, p < 0.05) at 20 kHz (Fig. 6B). The noise verapamil group was not significantly different when compared to the no noise saline and the no noise verapamil groups (Fig. 6B). One day after the noise exposure wave I amplitudes returned to normal for the noise saline group with no significant difference between the no noise groups (Fig. 6B). In addition, verapamil did not have any impact on wave I amplitude at later time points (Fig. 6B).

### The impact of Verapamil on central synchronous activity is dependent on noise exposure status

To investigate how L-type calcium channel inhibition affects central auditory synchrony under normal conditions versus following noise trauma, ABR Wave V amplitudes were examined in both noise-exposed and non-exposed animals treated with verapamil.

#### 12 kHz

**Fig. 7:**
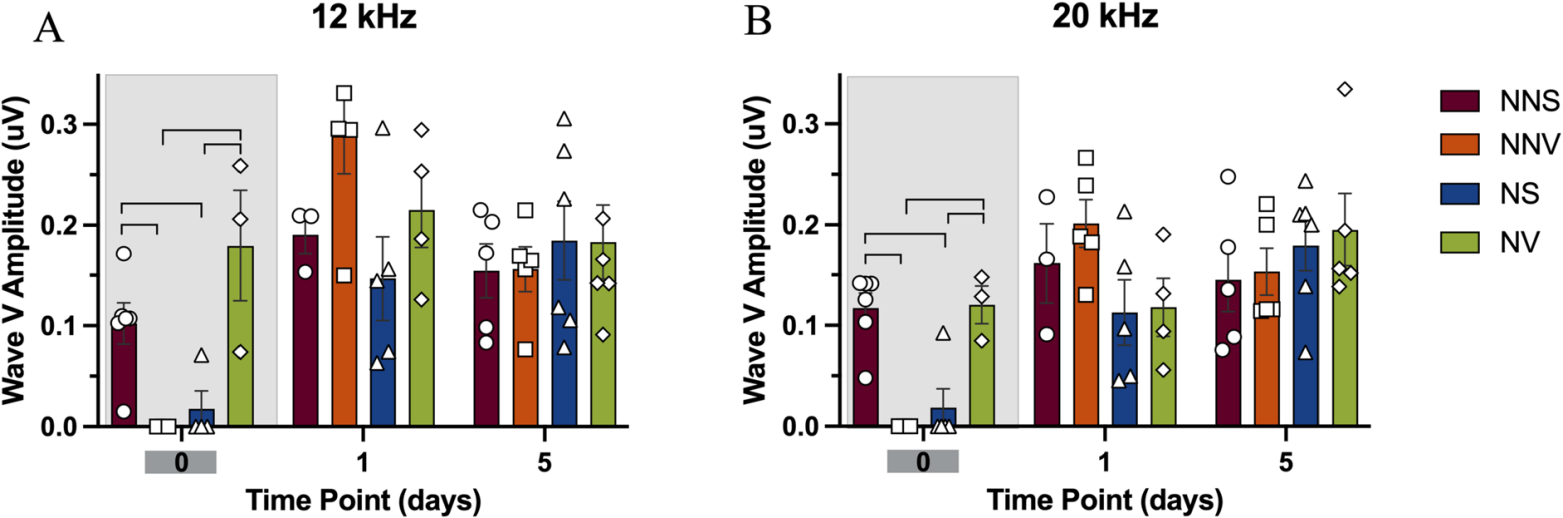
ABR wave V amplitude for auditory brainstem responses at 12 and 20 kHz. Wave V amplitudes on day zero, and one and five days after treatment and noise exposure are displayed. The wave I amplitudes were analyzed for no noise saline (circles—NNS), no noise verapamil (squares -NNV), noise saline (triangles—NS) and noise verapamil (diamonds—NV) groups at 12 kHz (**A**) and 20 kHz (**B**). Groups were compared using ANOVA with a post-hoc Tukey–Kramer test, and the error bars indicate the standard error of mean. Brackets denote significance at p < 0.05. Shaded set of bars indicate the time point at which significant changes were observed.

**Fig. 8:**
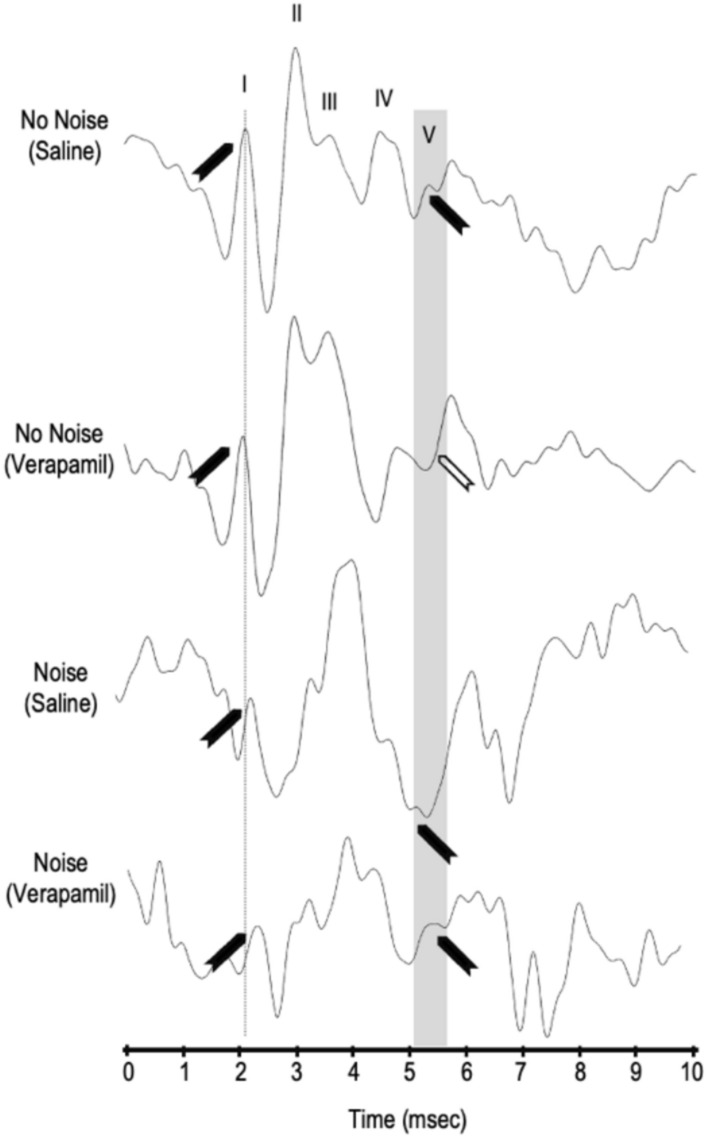
Auditory Brain Stem Response wave forms at 12 kHz at 80 dB SPL. Response to a tone pip at day 0 for no noise (saline or verapamil), and noise (saline or verapamil) groups are displayed. Wave I (auditory nerve), II (cochlear nucleus), III (superior olivary complex), IV (lateral lemniscus), V (inferior colliculus)^38,39^. are indicated on the no noise saline group. Wave I is indicated by a line and wave V is indicated by a gray bar. Waves that are present are marked by solid black arrowhead and waves that are absent are marked by a white arrowhead.

On day zero of noise exposure, there is a wave V amplitude reduction in the noise saline group compared to no noise saline group at 12 kHz (p < 0.05, Fig. 7A). In addition, no noise verapamil group had a non-detectable wave V amplitude on day zero of treatment (p < 0.05, Figs. 7A, 8). However, on day zero, there is no wave V amplitude reduction in the noise verapamil group compared to the noise saline and no noise verapamil group (p < 0.05, Fig. 7A). One day after the noise exposure, wave V amplitudes returned to normal for no noise verapamil and noise saline groups with no significant differences among the other groups (Fig. 7A). In addition, verapamil did not have any impact on wave V amplitude at later time points (Fig. 7A).

#### 20 kHz

On day zero of noise exposure there is a wave V amplitude reduction in the noise saline group compared to no noise saline group at 20 kHz (p < 0.05, Fig. 7B). In addition, no noise verapamil group had a non-detectable wave V amplitude on day zero of treatment (p < 0.05, Fig. 7B). However, on day zero of noise exposure, there is no wave V amplitude reduction in the noise verapamil group compared to the noise saline and no noise verapamil group (p < 0.05, Fig. 7B). One day after the noise exposure wave V amplitudes returned to normal for no noise verapamil and noise saline groups with no significant differences among the other groups (Fig. 7B). In addition, verapamil did not have any impact on wave V amplitude at later time points (Fig. 7B).

### Inhibition of the acoustic startle reflex occurs despite Verapamil-induced Wave V dys-synchrony

To further evaluate inferior colliculus activity during synchronous and dys-synchronous Wave V activity, gap inhibition of acoustic startle reflex (giASR) performance was assessed. Specifically, to determine the effects of Verapamil on startle inhibition performance, the degree of inhibition was evaluated with or without Verapamil in the noise and no noise groups.

#### G/SOS ASR Ratio at 45 dB

**Fig. 9:**
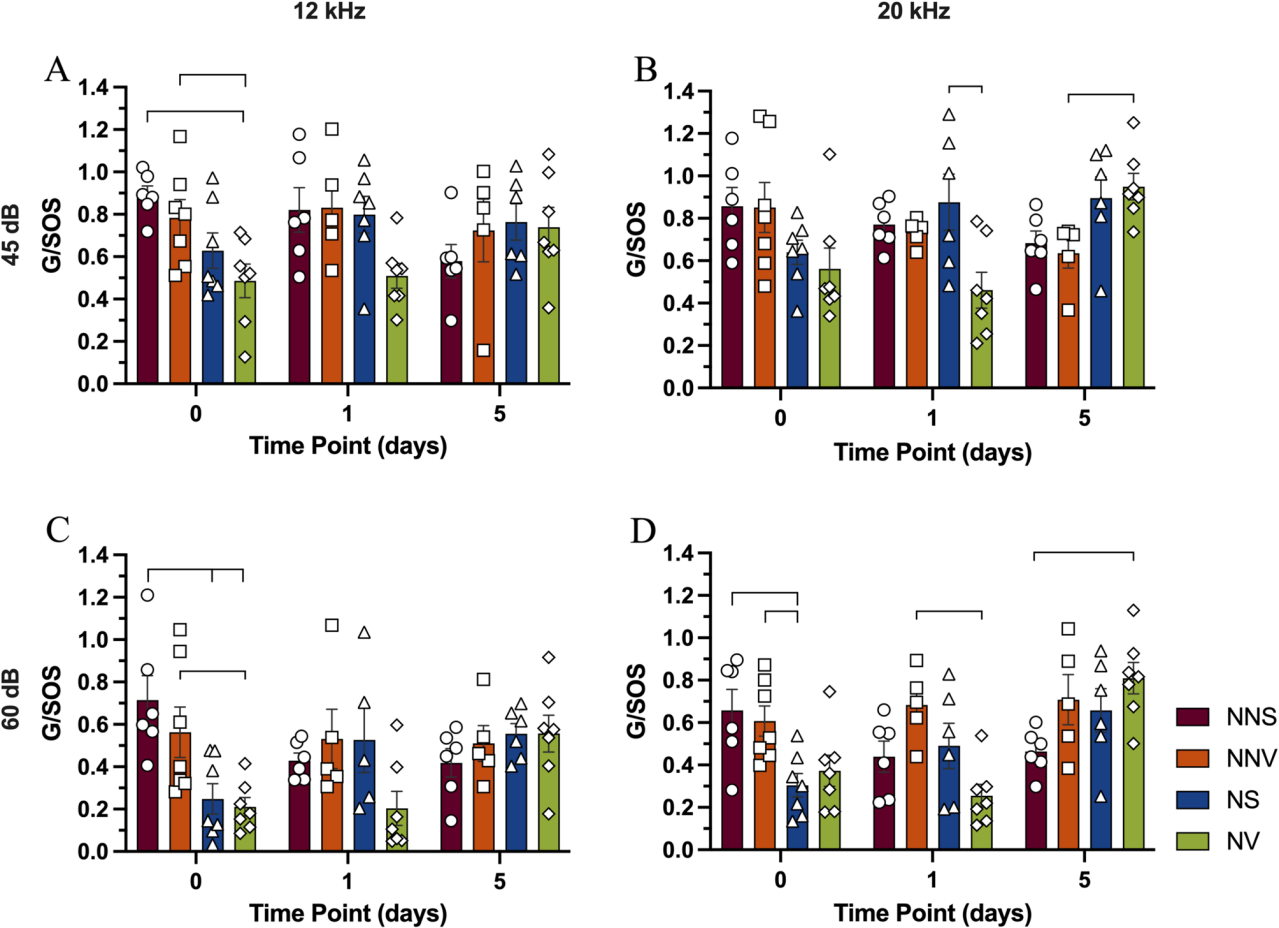
Gap to sound off startle ASR ratios at 12, and 20 at 45- and 60-dB SPL. The gap to sound off startle ASR ratios at three different time points, on day zero-, one- and five-day post treatment and noise exposure are displayed. The gap to sound off startle ASR ratios were analyzed for no noise saline (circles—NNS), no noise verapamil (squares—NNV), noise saline (triangles—NS) and noise verapamil (diamonds—NV) groups at 12 kHz (**A**, **C**), and 20 kHz (**B**, **D**) at 45 dB (**A**, **B**) and 60 dB (**C**, **D**). Groups were compared using ANOVA with a post-hoc Tukey–Kramer test, and the error bars indicate the standard error of mean. Brackets denote significance at p < 0.05.

On day zero of treatment, the noise verapamil group showed a decrease in G/SOS ratio compared to the no noise saline (46% 12 kHz p < 0.05) and no noise verapamil (38%, at 12 kHz p < 0.05) group on day zero of treatment (Fig. 9A). By one day after treatment, the noise verapamil group showed a decrease in G/SOS ratio compared to the noise saline group at 20 kHz (47%, p < 0.05, Fig. 9B). Five days after treatment noise verapamil group showed an increase in G/SOS ratio compared to the no noise verapamil group at 20 kHz (33%, p < 0.05, Fig. 9B).

#### G/SOS ASR Ratio at 60 dB

On day zero of treatment, the noise saline group showed a decrease in G/SOS ratio compared to the no noise saline group (65% and 54% at 12 and 20 kHz respectively p < 0.05 Fig. 9C-D) and no noise verapamil (50% at 20 p < 0.05, Fig. 9D). The noise verapamil group showed a decrease in G/SOS ratio compared to the no noise saline and no noise verapamil (70%, and 63% respectively, p < 0.05) groups on day zero of treatment at 12 kHz (Fig. 9C). By one day after treatment the noise verapamil group had significantly lower G/SOS ratios compared to no noise verapamil (63%, at 20 kHz, respectively, p < 0.05, Fig. 9D). Five days after treatment noise verapamil group showed deficits in G/SOS ratio compared to no noise saline group at 20 kHz (19%, p < 0.05, Fig. 9D).

### There is an inverse correlation between ABR Wave V amplitude and giASR performance

To evaluate the relationship between ABR Wave I and Wave V amplitude (synchrony) with the degree of inhibition during giASR testing (inferior colliculus function), correlations were performed at 12 and 20 kHz .

**Fig. 10:**
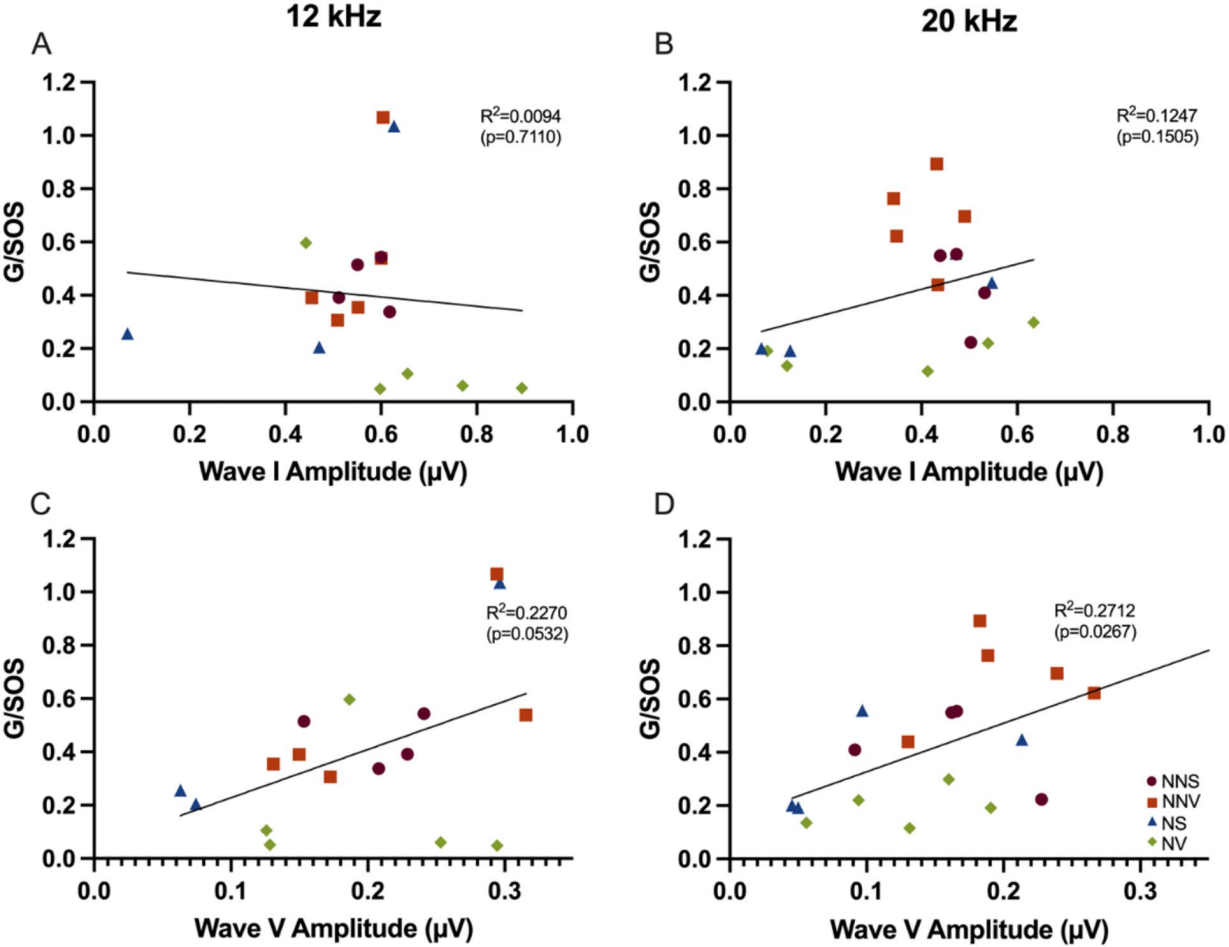
Startle response ratio G/SOS – 60 dB SPL in relation to ABR Wave I and V amplitude at 12 and 20 kHz. Correlation of Wave I amplitude (**A**-**B**) and Wave V amplitude (**C**-**D**) and giASR (G/SOS) are displayed. The correlation was assessed at one day across groups no noise saline (circle—NNS), no noise verapamil (square—NNV), noise saline (triangle—NS) and noise verapamil (diamonds—NV). Significance of the slopes was determined using a regression t-test; p-values are shown.

For ABR Wave I, there was no significant correlation between amplitude and giASR ratios (i.e., G/SOS) in response to 12 or 20 kHz, 45 dB SPL carrier tones (R^2^ = 0.0029 and 0.0801 respectively; data not shown). A similar result was found when the carrier tone was 12 or 20 kHz, 60 dB SPL (R^2^ = 0.0094 and 0.1247 respectively, Fig. 10A-B). While there was no significant correlation between ABR Wave V amplitudes and giASRs elicited with 12 or 20 kHz, 45 dB carrier tones (R^2^ = 0.2074 and 0.1916 respectively, data not shown), there was a significant correlation between ABR Wave V amplitudes and G/SOS in response to 12 or 20 kHz, 60 dB carrier tones (R^2^ = 0.2270 and 0.2712 respectively, *p* ≤ 0.05 Fig. 10C-D).

### Five days following noise exposure or Verapamil, central gain is enhanced

To assess changes in central auditory processing efficiency following noise exposure, the relationship between Wave I and Wave V amplitudes was analyzed to identify potential compensatory central gain mechanisms.

#### 12 kHz

**Fig. 11:**
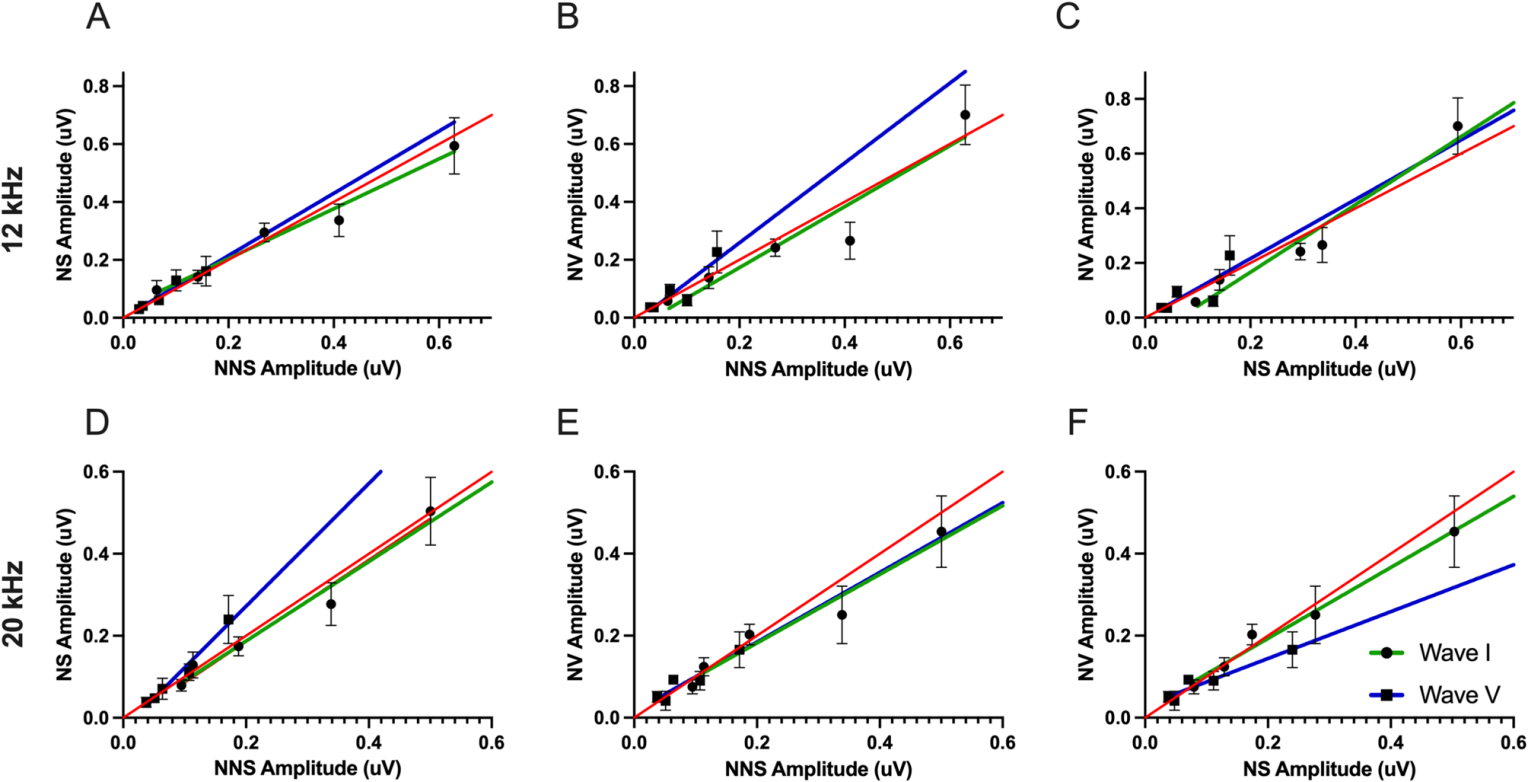
Tonal ABR wave amplitudes five days after treatment and noise exposure at 12 (A-C) and 20 kHz (D-F). Wave I (circles with green line) and wave V (squares with blue line) amplitudes for NS (**A**, **D**) and NV (**B**, **C**) vs. NNS. Additionally, NV vs NS wave I and wave V amplitudes are displayed (**C**, **F**). Red line represents the line of unity. Significance of the slopes was determined using a nonlinear fit from the line of unity; p-values are shown.

Five days following treatment and noise exposure the sound intensities evoked similar amplitudes for wave I and wave V in noise saline group compared to no noise saline group (the green and blue lines are not significantly different than the line of unity Fig. 11A). In addition, administration of verapamil prior to noise exposure did not change this effect, thus the sound intensities evoked similar amplitudes for wave I and wave V in the noise verapamil group compared to both the no noise saline and the noise saline group (the green and blue lines are not significantly different than the line of unity Fig. 11B and C respectively).

#### 20 kHz

Five days following treatment and noise exposure, the sound intensities evoked similar amplitudes for wave I in the noise saline group compared to the no noise saline group (the green line is not significantly different than the line of unity Fig. 11D). However, the sound intensities evoked higher amplitudes for wave V in noise saline group compared to no noise saline group (the blue line is significantly above the line of unity Fig. 11D, p = 0.0360). The sound intensities evoked similar amplitudes for wave I in the noise verapamil group compared to the no noise saline and noise saline groups (the green line is not significantly different than the line of unity Fig. 11 E and F respectively). In addition, administration of verapamil prior to noise exposure resulted in sound intensities evoking lower amplitudes for wave V in noise verapamil group compared to noise saline groups (the blue line is significantly below the line of unity Fig. 11F, p = 0.0219). Thus, the sound intensities evoked similar amplitudes for wave V in the noise verapamil group compared to the no noise saline group (the blue line is not significantly different than the line of unity Fig. 11E).

## Discussion

L-type calcium channels exhibit subtype-specific roles throughout the auditory pathway, with CaV1.3 channels governing transmitter release at inner-hair-cell ribbon synapses^11,12,14,30^., whereas CaV1.2 channels shape central synchrony in inferior-colliculus neurons^17,43^. The differential topographical distribution explains verapamil’s contrasting effects of peripheral protection versus central disruption, as the drug shows markedly different affinities for CaV1.3 (low affinity -micromolar) and CaV1.2 (high affinity -hundreds of micromolar) channels^20,25,44,45^. Pharmacological dissociation of synchronous and non-synchronous firing therefore provides a new window on the mechanisms that drive noise-induced auditory dysfunction^46^.

### Peripheral auditory protection

Verapamil may attenuate noise-induced reduction of ABR Wave I (Figs. 4, 6) by limiting CaV1.3-dependent calcium influx at the inner-hair-cell synapse^13,31^. Although CaV1.3 channels are relatively insensitive^20,44,45^, partial blockade appears sufficient to curb calcium-driven excitotoxicity, preventing swelling of afferents and synaptic loss after acoustic trauma^4,31,32,35^. Consequently, verapamil may limit cochlear Ca^2^⁺ overload and microvascular damage after acoustic trauma^4,35,47^. While preserving auditory-nerve synchrony despite persisting threshold shift, underscoring that L-type channels safeguard synaptic integrity rather than auditory sensitivity^48^.

### Central auditory effects

Systemic verapamil given to non-noise exposed animals produces an acute reduction in ABR Wave V amplitude (Figs. 5, 7, 8), consistent with transient suppression of inferior-colliculus gain that relies on tonically active L-type channels^43,49^. The high density of CaV1.2 currents in adult inferior-colliculus neurons^22,43^ accounts for this sensitivity, whereas rapid recovery of Wave V may reflect swift recruitment of P/Q-type CaV2.1 channels (insensitive to verapamil) that dominate mature auditory brainstem synapses^23,50^. Administered before noise exposure, verapamil preserves Wave V amplitude (Figs. 5, 7, 8), indicating that early L-type calcium entry is a trigger for central dys-synchrony^30^. Yet verapamil interferes with Ca^2^⁺-dependent gain control and synchronous firing in inferior colliculus neurons^43,49^.

### Dissociation of hyperactivity and synchrony

**Fig. 12:**
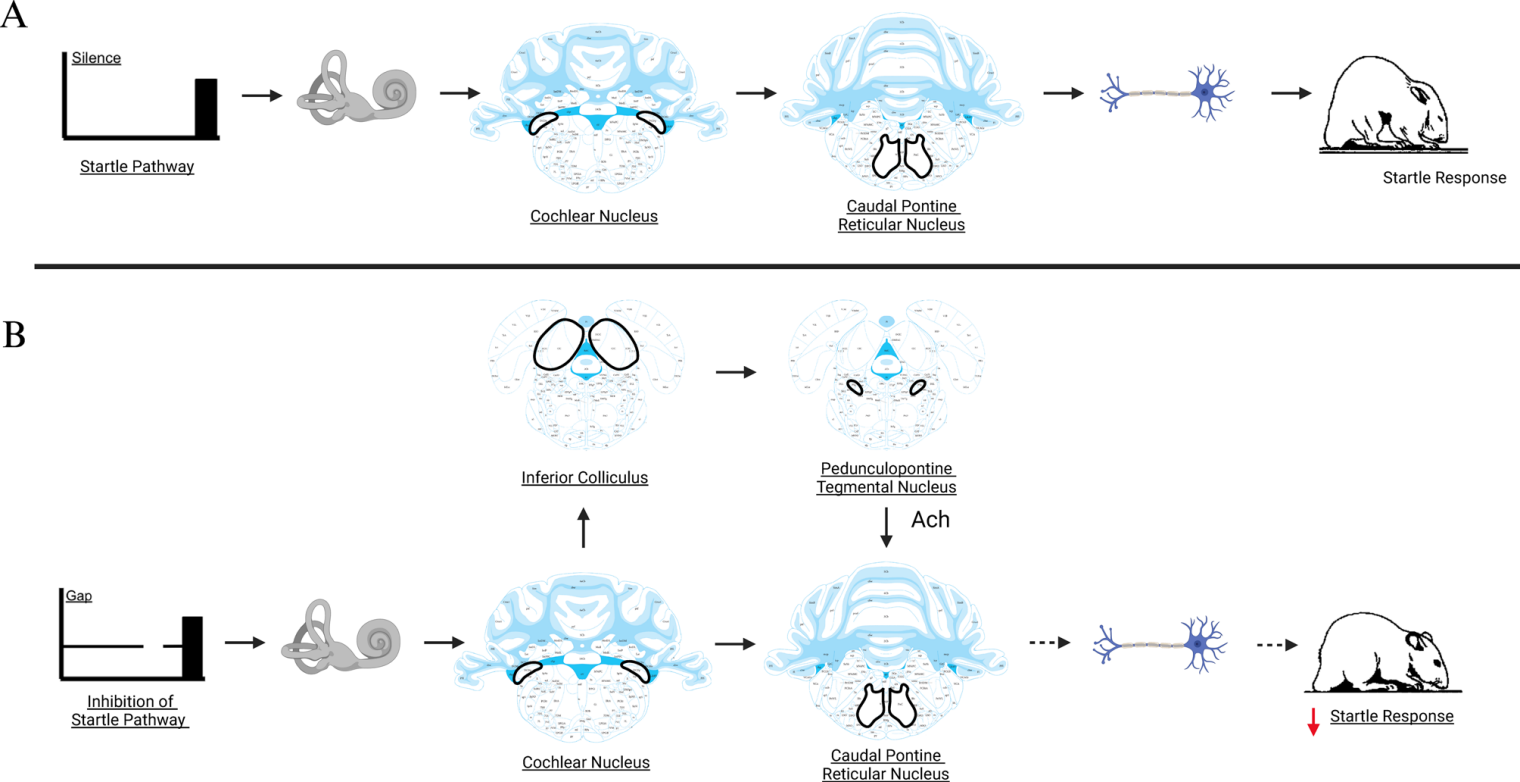
Acoustic startle reflex and gap inhibition of acoustic startle pathway. The startle reflex involves the activation of the caudal pontine reticular nucleus and subsequent activation of motor neurons (**A**). Gap inhibition of the acoustic startle reflex (giASR) engages the inferior colliculus (**B**). Increase of inferior colliculus activity increases the acetylcholine release from the pedunculopontine tegmental nucleus to the caudal pontine reticular nucleus and a subsequent decrease in motor neuron activity. This ultimately leads to inhibition of the acoustic startle reflex.

The most striking finding of our study is the clear dissociation between neuronal synchrony and hyperactivity in the inferior colliculus (Figs. 7, 9) following noise exposure and calcium channel inhibition. Multi-unit recordings reveal that inferior-colliculus hyperactivity persists even when synchronous ABR components are reduced^40,51^; lesion studies show that such hyperactivity can survive loss of dorsal-cochlear-nucleus drive^41,52^. Enhanced gap-inhibition of the acoustic startle reflex therefore co-exists with diminished Wave V amplitude (Figs. 7, 8, 9, 10, 11, 12), supporting prior evidence that hyperactivity and neural synchrony are mechanistically distinct phenomena. Specifically, noise trauma is known to produce concurrent increases in spontaneous neuronal firing rates and reduced synchronous activity in the auditory brainstem^42^, with timing-dependent neural plasticity and synchronization specifically disrupted independently of general neuronal activity^53^. The observed inverse correlation between Wave V amplitude and giASR performance (Figs. 10, 11) further supports this paradoxical state of increased activity co-occurring with decreased synchrony in inferior colliculus. The complex relationship between synchronous and non-synchronous neuronal activity demonstrates that these processes can be pharmacologically dissociated, providing important insights into the mechanisms underlying noise-induced auditory dysfunction^30,46^.

### Central gain and adaptive mechanisms

Selective modulation of L-type channels demonstrates that neuronal synchrony and hyperexcitability are separable dimensions of auditory coding^54,55^. Similar central-gain adaptations have been described after cochlear synaptopathy^46,56^ and work in the dorsal cochlear nucleus points to homeostatic re-balancing of excitation and inhibition as an underlying mechanism^57–59^. Because P/Q-type release machinery remains unaffected^23,50^, verapamil may curb maladaptive central gain while sparing essential temporal processing, highlighting the therapeutic promise of subtype-targeted calcium-channel modulators^19,60^.

The prevention of noise-induced central gain by verapamil administration (Fig. 7), reveals another important aspect of calcium channel function in auditory processing. Under normal circumstances, noise exposure triggers compensatory increases in central auditory responsiveness that may contribute to hyperacusis and other auditory processing disorders^61,62^. Verapamil’s ability to dampen this maladaptive central gain while preserving peripheral function suggests that L-type calcium channels play a crucial role in the plastic changes that follow acoustic trauma^63,64^. This finding has important implications for understanding how the auditory system attempts to compensate for peripheral damage and how pharmaceutical interventions might be used to prevent harmful adaptations while preserving beneficial ones^65–67^.

### Study limitations and future directions

Several important limitations must be acknowledged in interpreting these results. The small sample sizes, particularly for critical comparisons involving the no noise verapamil group, limit statistical power and the ability to generalize findings. The single-dose, acute administration protocol, while appropriate for demonstrating proof-of-concept, may not reflect optimal therapeutic dosing strategies. The relatively high verapamil dose employed, while within established experimental ranges^68^, may produce off-target effects that complicate interpretation of results^10,69^.

Future studies could employ dose–response protocols, larger sample sizes, and subtype-selective calcium channel antagonists to better characterize the specific contributions of CaV1.2 versus CaV1.3 channels to auditory function^70,71^. Additionally, direct measurement of the expression and distribution of alternative calcium receptors and calcium imaging studies would help validate the proposed compensatory mechanisms and their role in maintaining inferior colliculus hyperactivity during L-type channel blockade^15,72,73^.

## Conclusions

This study demonstrates that L-type voltage-gated calcium channels differentially contribute to peripheral and central auditory function through subtype-specific mechanisms^21,24^. L-type calcium channel inhibition reveals the complex relationship between synchronous and non-synchronous neuronal activity in the auditory system, showing that these processes can be pharmacologically dissociated^16,74^. The findings suggest that calcium channel dysfunction may represent an underlying mechanism for disorders involving altered auditory synchrony^75,76^, while compensatory pathways may maintain hyperactivity states^77,78^. Understanding these relationships enhances our knowledge of noise-induced auditory dysfunction mechanisms and supports the development of targeted therapeutic approaches for auditory disorders involving disrupted calcium homeostasis^79^.

## Data Availability

The datasets generated during and/or analyzed during the current study are available from the corresponding author on reasonable request.

## References

[1] Mitchell, R. E. How many deaf people are there in the United States? estimates from the survey of income and program participation. *J. Deaf. Stud. Deaf. Educ.* **11**(1), 112–119 (2006). 16177267 10.1093/deafed/enj004

[2] Blazer, D. G., Domnitz, S. & Liverman, C. T. Hearing health care for adults: Priorities for improving access and affordability. In National Academies of Sciences, Engineering, and Medicine. Washington, DC: The National Academies Press (2016). 27280276

[3] Helzner, E. P. et al. Race and sex differences in age-related hearing loss: the Health, Aging and Body Composition Study. *J. Am. Geriatr. Soc.* **53**(12), 2119–2127 (2005). 16398896 10.1111/j.1532-5415.2005.00525.x

[4] Kujawa, S. G. & Liberman, M. C. Adding insult to injury: cochlear nerve degeneration after “temporary” noise-induced hearing loss. *J. Neurosci.* **29**(45), 14077–14085 (2009). 19906956 10.1523/JNEUROSCI.2845-09.2009 PMC2812055

[5] Mulders, W. H. & Robertson, D. Hyperactivity in the auditory midbrain after acoustic trauma: dependence on cochlear activity. *Neuroscience* **164**(2), 733–746 (2009). 19699277 10.1016/j.neuroscience.2009.08.036

[6] Kaltenbach, J. A. & Zhang, J. Intense sound-induced plasticity in the dorsal cochlear nucleus of rats: evidence for cholinergic receptor upregulation. *Hear Res.* **226**(1–2), 232–243 (2007). 16914276 10.1016/j.heares.2006.07.001

[7] Dolphin, A. C. & Lee, A. Presynaptic calcium channels: specialized control of synaptic neurotransmitter release. *Nat. Rev. Neurosci.* **21**(4), 213–229 (2020). 32161339 10.1038/s41583-020-0278-2 PMC7873717

[8] Scarnati, M. S. et al. Presynaptic calcium channel open probability and changes in calcium influx throughout the action potential determined using ap-waveforms. *Front. Synaptic. Neurosci.* **12**, 17 (2020). 32425764 10.3389/fnsyn.2020.00017 PMC7212394

[9] Clarke, S. G., Scarnati, M. S. & Paradiso, K. G. Neurotransmitter release can be stabilized by a mechanism that prevents voltage changes near the end of action potentials from affecting calcium currents. *J. Neurosci.* **36**(45), 11559–11572 (2016). 27911759 10.1523/JNEUROSCI.0066-16.2016 PMC5125219

[10] Striessnig, J. et al. Role of voltage-gated L-type Ca2+ channel isoforms for brain function. *Biochem. Soc. Trans.* **34**(Pt 5), 903–909 (2006). 17052224 10.1042/BST0340903

[11] Brandt, A., Striessnig, J. & Moser, T. CaV1.3 channels are essential for development and presynaptic activity of cochlear inner hair cells. *J. Neurosci.* **23**(34), 10832–40 (2003). 14645476 10.1523/JNEUROSCI.23-34-10832.2003 PMC6740966

[12] Sheets, L., Kindt, K. S. & Nicolson, T. Presynaptic CaV1.3 channels regulate synaptic ribbon size and are required for synaptic maintenance in sensory hair cells. *J. Neurosci.* **32**(48), 17273–86 (2012). 23197719 10.1523/JNEUROSCI.3005-12.2012 PMC3718275

[13] Brandt, A., Khimich, D. & Moser, T. Few CaV1.3 channels regulate the exocytosis of a synaptic vesicle at the hair cell ribbon synapse. *J. Neurosci.* **25**(50), 11577–85 (2005). 16354915 10.1523/JNEUROSCI.3411-05.2005 PMC6726013

[14] Platzer, J. et al. Congenital deafness and sinoatrial node dysfunction in mice lacking class D L-type Ca2+ channels. *Cell* **102**(1), 89–97 (2000). 10929716 10.1016/s0092-8674(00)00013-1

[15] Zhang, S. Y. et al. Role of L-type Ca(2+) channels in transmitter release from mammalian inner hair cells I Gross Sound-evoked potentials. *J Neurophysiol.* **82**(6), 3307–3315 (1999). 10601462 10.1152/jn.1999.82.6.3307

[16] Nouvian, R. et al. Structure and function of the hair cell ribbon synapse. *J. Membr. Biol.* **209**(2–3), 153–165 (2006). 16773499 10.1007/s00232-005-0854-4 PMC1764598

[17] Ebbers, L. et al. L-type calcium channel Cav1.2 is required for maintenance of auditory brainstem nuclei. *J. Biol. Chem.* **290**(39), 23692–710 (2015). 26242732 10.1074/jbc.M115.672675 PMC4583033

[18] Zuccotti, A. et al. L-type CaV1.2 deletion in the cochlea but not in the brainstem reduces noise vulnerability: implication for CaV1.2-mediated control of cochlear BDNF expression. *Front. Mol. Neurosci.* **6**, 20 (2013). 23950737 10.3389/fnmol.2013.00020 PMC3739414

[19] Nanou, E. & Catterall, W. A. Calcium channels, synaptic plasticity, and neuropsychiatric disease. *Neuron* **98**(3), 466–481 (2018). 29723500 10.1016/j.neuron.2018.03.017

[20] Tarabova, B., Lacinova, L. & Engel, J. Effects of phenylalkylamines and benzothiazepines on Ca(v)1.3-mediated Ca2+ currents in neonatal mouse inner hair cells. *Eur J Pharmacol.* **573**(1–3), 39–48 (2007). 17651721 10.1016/j.ejphar.2007.06.050

[21] Young, S. M. Jr. & Veeraraghavan, P. Presynaptic voltage-gated calcium channels in the auditory brainstem. *Mol. Cell Neurosci.* **112**, 103609 (2021). 33662542 10.1016/j.mcn.2021.103609 PMC8085099

[22] Iwasaki, S. et al. Developmental changes in calcium channel types mediating central synaptic transmission. *J. Neurosci.* **20**(1), 59–65 (2000). 10627581 10.1523/JNEUROSCI.20-01-00059.2000 PMC6774098

[23] Lubbert, M. et al. Ca(V)2.1 alpha(1) Subunit Expression Regulates Presynaptic Ca(V)2.1 Abundance and Synaptic Strength at a Central Synapse. *Neuron.* **101**(2), 260–273 (2019). 30545599 10.1016/j.neuron.2018.11.028 PMC6413316

[24] Wadel, K., Neher, E. & Sakaba, T. The coupling between synaptic vesicles and Ca2+ channels determines fast neurotransmitter release. *Neuron* **53**(4), 563–575 (2007). 17296557 10.1016/j.neuron.2007.01.021

[25] Cai, D., Mulle, J. G. & Yue, D. T. Inhibition of recombinant Ca2+ channels by benzothiazepines and phenylalkylamines: class-specific pharmacology and underlying molecular determinants. *Mol. Pharmacol.* **51**(5), 872–881 (1997). 9145926

[26] Bergson, P. et al. Verapamil block of T-type calcium channels. *Mol. Pharmacol.* **79**(3), 411–419 (2011). 21149638 10.1124/mol.110.069492 PMC3061365

[27] Li, W. & Shi, G. How Ca(V)1.2-bound verapamil blocks Ca(2+) influx into cardiomyocyte: Atomic level views. *Pharmacol. Res.* **139**, 153–157 (2019). 30447294 10.1016/j.phrs.2018.11.017

[28] Orrenius, S., Zhivotovsky, B. & Nicotera, P. Regulation of cell death: the calcium-apoptosis link. *Nat. Rev. Mol. Cell Biol.* **4**(7), 552–565 (2003). 12838338 10.1038/nrm1150

[29] Szydlowska, K. & Tymianski, M. Calcium, ischemia and excitotoxicity. *Cell Calcium.* **47**(2), 122–129 (2010). 20167368 10.1016/j.ceca.2010.01.003

[30] Hu, N., Rutherford, M. A. & Green, S. H. Protection of cochlear synapses from noise-induced excitotoxic trauma by blockade of Ca(2+)-permeable AMPA receptors. *Proc. Natl. Acad. Sci. U S A.* **117**(7), 3828–3838 (2020). 32015128 10.1073/pnas.1914247117 PMC7035499

[31] Blum, K. et al. Noise-induced cochlear synaptopathy in C57BL/6 N mice as a function of trauma strength: ribbons are more vulnerable than postsynapses. *Front. Cell Neurosci.* **18**, 1465216 (2024). 39411002 10.3389/fncel.2024.1465216 PMC11473312

[32] Zhao, H. B., Zhu, Y. & Liu, L. M. Excess extracellular K(+) causes inner hair cell ribbon synapse degeneration. *Commun. Biol.* **4**(1), 24 (2021). 33398038 10.1038/s42003-020-01532-w PMC7782724

[33] Liu, J. et al. Interaction of a calcium channel blocker with noise in cochlear function in guinea pig. *Acta Otolaryngol.* **132**(11), 1140–1144 (2012). 22780109 10.3109/00016489.2012.690534

[34] Uemaetomari, I. et al. L-type voltage-gated calcium channel is involved in the pathogenesis of acoustic injury in the cochlea. *Tohoku J. Exp. Med.* **218**(1), 41–47 (2009). 19398872 10.1620/tjem.218.41

[35] Goldwyn, B. G. & Quirk, W. S. Calcium channel blockade reduces noise-induced vascular permeability in cochlear stria vascularis. *Laryngoscope* **107**(8), 1112–1116 (1997). 9261017 10.1097/00005537-199708000-00019

[36] Bobbin, R. P. et al. Nimodipine, an L-channel Ca2+ antagonist, reverses the negative summating potential recorded from the guinea pig cochlea. *Hear Res.* **46**(3), 277–287 (1990). 2168361 10.1016/0378-5955(90)90009-e

[37] Berlin, C. I. et al. Auditory neuropathy/dys-synchrony: diagnosis and management. *Ment. Retard Dev. Disabil. Res. Rev.* **9**(4), 225–231 (2003). 14648814 10.1002/mrdd.10084

[38] Funai, H. & Funasaka, S. Experimental study on the effect of inferior colliculus lesions upon auditory brain stem response. *Audiology* **22**(1), 9–19 (1983). 6830534 10.3109/00206098309072766

[39] Zhou, X. et al. Auditory brainstem responses in 10 inbred strains of mice. *Brain Res.* **1091**(1), 16–26 (2006). 16516865 10.1016/j.brainres.2006.01.107 PMC2859191

[40] Manzoor, N. F. et al. Noise-induced hyperactivity in the inferior colliculus: its relationship with hyperactivity in the dorsal cochlear nucleus. *J. Neurophysiol.* **108**(4), 976–988 (2012). 22552192 10.1152/jn.00833.2011 PMC3424082

[41] Kaltenbach, J. A. & Afman, C. E. Hyperactivity in the dorsal cochlear nucleus after intense sound exposure and its resemblance to tone-evoked activity: a physiological model for tinnitus. *Hear Res.* **140**(1–2), 165–172 (2000). 10675644 10.1016/s0378-5955(99)00197-5

[42] Schrode, K.M. et al. Central compensation in auditory brainstem after damaging noise exposure. *eNeuro*. **5**(4), (2018). 10.1523/ENEURO.0250-18.2018 PMC6096756 30123822

[43] N’Gouemo, P., Faingold, C. L. & Morad, M. Calcium channel dysfunction in inferior colliculus neurons of the genetically epilepsy-prone rat. *Neuropharmacology* **56**(3), 665–675 (2009). 19084544 10.1016/j.neuropharm.2008.11.005 PMC2638996

[44] Hockerman, G. H. et al. Molecular determinants of high affinity phenylalkylamine block of L-type calcium channels in transmembrane segment IIIS6 and the pore region of the alpha1 subunit. *J. Biol. Chem.* **272**(30), 18759–18765 (1997). 9228049 10.1074/jbc.272.30.18759

[45] Hockerman, G. H. et al. Molecular determinants of high affinity phenylalkylamine block of L-type calcium channels. *J. Biol. Chem.* **270**(38), 22119–22122 (1995). 7673189 10.1074/jbc.270.38.22119

[46] Lobarinas, E., Spankovich, C. & Le Prell, C. G. Evidence of “hidden hearing loss” following noise exposures that produce robust TTS and ABR wave-I amplitude reductions. *Hear Res.* **349**, 155–163 (2017). 28003148 10.1016/j.heares.2016.12.009

[47] Saddala, M. S. et al. Discovery of novel L-type voltage-gated calcium channel blockers and application for the prevention of inflammation and angiogenesis. *J. Neuroinflam.* **17**(1), 132 (2020). 10.1186/s12974-020-01801-9 PMC7183139 32334630

[48] Miko, I. J. & Sanes, D. H. Transient gain adjustment in the inferior colliculus is serotonin- and calcium-dependent. *Hear Res.* **251**(1–2), 39–50 (2009). 19232535 10.1016/j.heares.2009.02.003 PMC2670942

[49] Sivaramakrishnan, S. & Oliver, D. L. Distinct K currents result in physiologically distinct cell types in the inferior colliculus of the rat. *J. Neurosci.* **21**(8), 2861–2877 (2001). 11306638 10.1523/JNEUROSCI.21-08-02861.2001 PMC6762543

[50] Heeringa, A. N. & van Dijk, P. The dissimilar time course of temporary threshold shifts and reduction of inhibition in the inferior colliculus following intense sound exposure. *Hear Res.* **312**, 38–47 (2014). 24650953 10.1016/j.heares.2014.03.004

[51] Faingold, C. L. et al. Inferior colliculus neuronal response abnormalities in genetically epilepsy-prone rats: evidence for a deficit of inhibition. *Life Sci.* **39**(10), 869–878 (1986). 3747711 10.1016/0024-3205(86)90368-1

[52] Land, R., Burghard, A. & Kral, A. The contribution of inferior colliculus activity to the auditory brainstem response (ABR) in mice. *Hear Res.* **341**, 109–118 (2016). 27562195 10.1016/j.heares.2016.08.008

[53] Koehler, S. D. & Shore, S. E. Stimulus timing-dependent plasticity in dorsal cochlear nucleus is altered in tinnitus. *J. Neurosci.* **33**(50), 19647–19656 (2013). 24336728 10.1523/JNEUROSCI.2788-13.2013 PMC3858633

[54] Li, S., Choi, V. & Tzounopoulos, T. Pathogenic plasticity of Kv7.2/3 channel activity is essential for the induction of tinnitus. *Proc. Natl. Acad. Sci. U S A.* **110**(24), 9980–5 (2013). 23716673 10.1073/pnas.1302770110 PMC3683764

[55] Pilati, N. et al. Mechanisms contributing to central excitability changes during hearing loss. *Proc. Natl. Acad. Sci. U S A.* **109**(21), 8292–8297 (2012). 22566618 10.1073/pnas.1116981109 PMC3361412

[56] Shore, S. E. et al. Dorsal cochlear nucleus responses to somatosensory stimulation are enhanced after noise-induced hearing loss. *Eur. J. Neurosci.* **27**(1), 155–168 (2008). 18184319 10.1111/j.1460-9568.2007.05983.x PMC2614620

[57] Wickesberg, R. E. & Oertel, D. Delayed, frequency-specific inhibition in the cochlear nuclei of mice: a mechanism for monaural echo suppression. *J. Neurosci.* **10**(6), 1762–1768 (1990). 1972392 10.1523/JNEUROSCI.10-06-01762.1990 PMC6570299

[58] Tzounopoulos, T. Mechanisms of synaptic plasticity in the dorsal cochlear nucleus: plasticity-induced changes that could underlie tinnitus. *Am. J. Audiol.* **17**(2), S170–S175 (2008). 18978197 10.1044/1059-0889(2008/07-0030) PMC2804917

[59] Catterall, W. A., Leal, K. & Nanou, E. Calcium channels and short-term synaptic plasticity. *J. Biol. Chem.* **288**(15), 10742–10749 (2013). 23400776 10.1074/jbc.R112.411645 PMC3624454

[60] Auerbach, B. D., Rodrigues, P. V. & Salvi, R. J. Central gain control in tinnitus and hyperacusis. *Front. Neurol.* **5**, 206 (2014). 25386157 10.3389/fneur.2014.00206 PMC4208401

[61] Chen, G. D. et al. Salicylate-induced cochlear impairments, cortical hyperactivity and re-tuning, and tinnitus. *Hear Res.* **295**, 100–113 (2013). 23201030 10.1016/j.heares.2012.11.016 PMC4191647

[62] Norena, A. J. An integrative model of tinnitus based on a central gain controlling neural sensitivity. *Neurosci. Biobehav. Rev.* **35**(5), 1089–1109 (2011). 21094182 10.1016/j.neubiorev.2010.11.003

[63] Schaette, R. & Kempter, R. Development of tinnitus-related neuronal hyperactivity through homeostatic plasticity after hearing loss: a computational model. *Eur. J. Neurosci.* **23**(11), 3124–3138 (2006). 16820003 10.1111/j.1460-9568.2006.04774.x

[64] Turner, J. G. et al. Gap detection deficits in rats with tinnitus: a potential novel screening tool. *Behav. Neurosci.* **120**(1), 188–195 (2006). 16492129 10.1037/0735-7044.120.1.188

[65] Wang, H. et al. Plasticity at glycinergic synapses in dorsal cochlear nucleus of rats with behavioral evidence of tinnitus. *Neuroscience* **164**(2), 747–759 (2009). 19699270 10.1016/j.neuroscience.2009.08.026 PMC2761999

[66] Le Prell, C. G. et al. Mechanisms of noise-induced hearing loss indicate multiple methods of prevention. *Hear Res.* **226**(1–2), 22–43 (2007). 17141991 10.1016/j.heares.2006.10.006 PMC1995566

[67] Zhou, X. & Merzenich, M. M. Environmental noise exposure degrades normal listening processes. *Nat. Commun.* **3**, 843 (2012). 22588305 10.1038/ncomms1849

[68] Freeze, B. S., McNulty, M. M. & Hanck, D. A. State-dependent verapamil block of the cloned human Ca(v)3.1 T-type Ca(2+) channel. *Mol. Pharmacol.* **70**(2), 718–26 (2006). 16699084 10.1124/mol.106.023473

[69] Berrout, J. & Isokawa, M. Homeostatic and stimulus-induced coupling of the L-type Ca2+ channel to the ryanodine receptor in the hippocampal neuron in slices. *Cell Calcium* **46**(1), 30–38 (2009). 19411104 10.1016/j.ceca.2009.03.018 PMC2703683

[70] Koschak, A. et al. Alpha 1D (Cav1.3) subunits can form l-type Ca2+ channels activating at negative voltages. *J. Biol. Chem.* **276**(25), 22100–6 (2001). 11285265 10.1074/jbc.M101469200

[71] Matsuo, N. et al. Comprehensive behavioral phenotyping of ryanodine receptor type 3 (RyR3) knockout mice: decreased social contact duration in two social interaction tests. *Front. Behav. Neurosci.* **3**, 3 (2009). 19503748 10.3389/neuro.08.003.2009 PMC2691151

[72] Sukhareva, M. et al. Functional properties of ryanodine receptors in hippocampal neurons change during early differentiation in culture. *J. Neurophysiol.* **88**(3), 1077–1087 (2002). 12205130 10.1152/jn.2002.88.3.1077

[73] Bull, R. et al. Ischemia enhances activation by Ca2+ and redox modification of ryanodine receptor channels from rat brain cortex. *J. Neurosci.* **28**(38), 9463–9472 (2008). 18799678 10.1523/JNEUROSCI.2286-08.2008 PMC6671122

[74] Schaette, R. & McAlpine, D. Tinnitus with a normal audiogram: physiological evidence for hidden hearing loss and computational model. *J. Neurosci.* **31**(38), 13452–13457 (2011). 21940438 10.1523/JNEUROSCI.2156-11.2011 PMC6623281

[75] Guest, H. et al. Tinnitus with a normal audiogram: Relation to noise exposure but no evidence for cochlear synaptopathy. *Hear Res.* **344**, 265–274 (2017). 27964937 10.1016/j.heares.2016.12.002 PMC5256478

[76] Chikamori, Y. et al. Locus coeruleus-induced inhibition of dorsal cochlear nucleus neurons in comparison with lateral vestibular nucleus neurons. *Brain Res.* **194**(1), 53–63 (1980). 7378847 10.1016/0006-8993(80)91318-9

[77] Kaltenbach, J. A. Tinnitus: Models and mechanisms. *Hear Res.* **276**(1–2), 52–60 (2011). 21146597 10.1016/j.heares.2010.12.003 PMC3109239

[78] Washnik, N. J. et al. Evaluation of cochlear activity in normal-hearing musicians. *Hear Res.* **395**, 108027 (2020). 32659614 10.1016/j.heares.2020.108027 PMC7483999

[79] Bramhall, N. F. Use of the auditory brainstem response for assessment of cochlear synaptopathy in humans. *J. Acoust. Soc. Am.* **150**(6), 4440 (2021). 34972291 10.1121/10.0007484 PMC10880747

